# Biotechnology, Bioinformatics and Bioinformation in an Autobiography

**DOI:** 10.6026/97320630016039

**Published:** 2020-01-31

**Authors:** Pandjassarame Kangueane

**Affiliations:** 1 Biomedical Informatics Private Limited, Irulan Sandy Annex, Pondicherry 607 402

**Keywords:** Biotechnology, Bioinformatics, Bioinformation, Teaching, Research, Farming, Editing, Enzymes, Lipase, cry toxins, HLA typing, PPI, Epitope design, Genome analysis, Exons, Introns, Gene fusion, Books, Journalism, Social Responsibility, Business, Meetings, Conferences, Academic Freedom, Peers, Students, Scientists, AFP, Citations, h Index, Research Impact

## Abstract

Science is observation. Application of Science is engineering. A 0% error is desired in Science, while a 25% error is usually allowed in Engineering. Technology is engineering with Science where the error rate is considerably reduced to improve precision. Biotechnology is truly interdisciplinary with an optimal mix of physics, chemistry and biology linked by Mathematics. Chemistry evolved into Chemical Engineering and thus Biochemistry into Biochemical Engineering. Biochemical engineering with genetics and molecular biology created Biotechnology. Biotechnology with computer science developed Bioinformatics. Bioinformatics used biological data to glean BIOINFORMATION for Biological Knowledge Discovery (BKD). This helped to accelerate drug discovery and develop other biologics (biomarkers, vaccines, seed developments, bio-fertilizers and bio-pesticides) towards improved service in healthcare, agriculture, food production, food processing and food distribution across international borders as per demand supply in the supply chain. It is joyful to realize the personal experience with the multifaceted features of Biotechnology, Bioinformatics and Bioinformation in a comprehensive manner over a period of three decades. This educational path is truly exciting, engaging and enterprising. This journey provided an opportunity to debate on cry toxins, lipase, ibuprofen, HLA alleles, antigens, peptide vaccines, protein-protein interactions, genomes and biological knowledge discovery models.

## Science at School:

“Mother, Father, Teacher and God: This is the order of priority in human life

I was born at Cuddalore (a British colony during pre-independence India) and lived at Pondicherry (a French Territory). I did my schooling at Petit Seminarie Higher secondary School at Pondicherry which is a two century old school started during the French occupation in India. During school I was interested in science. Moreover, I was intrigued by the valency of carbon and even further by hydro carbons. This is how it started. Thus, a simple interest in hydrocarbons under the prescription of organic chemistry at school got blown up to analyse protein-protein interfaces made up of amino acids held by non-covalent bonds over a period of 3 decades. Therefore, it is of interest to document several important moments during this path.

“Linking advancement to science in an autobiography during a lifetime of a scientist”

I grew up in a slow moving Union Territory, Pondicherry, India doing my schooling. Then, I moved to the fast moving Chennai, Tamilnadu for my undergraduate education in Industrial Biotechnology at AC college of Technology, Anna University, Chennai, Tamilnadu, India. This place showed the passion for science.

“Incremental advancement to science is a slow process”

## Industrial Biotechnology at AC college of Technology, Anna University:

I completed my undergraduate degree (B. Tech in Industrial Biotechnology) from Alagappa Chettiar college of Technology (ACTech) at Anna University (AU), Chennai, Tamilnadu, India. ACTech provided courses in Chemical Engineering, Leather Technology, Textile Technology and Industrial Biotechnology. The contribution of Chemical Engineering, Leather Technology, Textile Technology and Industrial Biotechnology industries to India’s GDP is about 20% highlighting its significance. The role played by ACTech in National growth is highly considerable. Several Chemical, Biotechnology, Leather and Textile industries have links with ACTech and most of them were founded by Alumni members of ACTech. Thanks to these developments, India witnessed an industrial revolution in late 90’s.

Industrial Biotechnology curriculum at Anna University is truly interdisciplinary consisting of subjects in Engineering, Technology and Biology. A perfect blend of chemical engineering and with modern biology was designed at ACTech. This is not possible without (Late) Kunthala Jayaraman (KJ). She is the mother of Industrial Biotechnology Education in the world. She founded the Centre for Biotechnology (CBT) at ACTech, Anna University [1]. She created a curriculum for Industrial Biotechnology with an optimal blend of Science, Engineering and Technology [2]. This is not possible without discussion, debate and collaboration across various departments at Anna University. The curriculum was novel with mathematics, physics, chemistry and biology. We were the second batch of students experiencing the syllabus in real time. We were shuffling between departments in civil, mechanical, electrical, computer and chemical engineering in addition to molecular biology related laboratories. Thus, CBT at AU made significant contribution to human resource development in Industrial Biotechnology in serving humanity across continents. CBT was busy with several visiting scientists of distinction such as Werner Arber (noble laureate for the discovery of restriction enzymes), Thomas Nutman and several others. This is not even imaginable without KJ. She was extremely active with bubbling ideas.

KJ was an able leader in Biotechnology. She is an Indian Biotechnologist of eminence. She is a women scientist of wonders. She established several AU-NIH (Indo-USA) and AU-ETH (Indo-Swiss) projects with mutual benefits through collaborations. She did not miss the technology aspect in all her projects and collaborations. She was extraordinary in entrepreneurial spirit. She strengthened University-Industry interactions through several initiatives. She was serious about the bio pesticides, filariasis and tuberculosis of relevance to the sub-continent [3] - [14]. She was also keen on optimization and scale up projects installing bioreactors. She established the AU-SPIC bioprocess laboratory at Tharamani, Chennai, India. She was successful in creating corpus funds to support new faculty members. She was the founding Director. Later, she became Dean of Technology, ACTech, AU. She then joined the Vellore Institute of Technology, Vellore, Tamilnadu, India to further promote Biotechnology during her last years of life. She left us on December 13, 2010 leaving behind several professional memories. The scientific community truly misses her. Her contribution to Indian Biotechnology and Anna University is highly significant.

## Bacillus thuringiensis cry toxins production in co-culture:

During the summer of 1994, after a small stint with the research and development section (yeast and tablet formulation) at TTK Pharma Limited (Pallavaram Branch), Chennai, Tamilnadu, India, I started working on Bacillus thuriengis (B.t). We (P. Kangueane, G. Kalaiselvi and R. Sachidanandam) were trying to estimate the individual population dynamics of B.t.a (Bacillus thuriengis subspecies aizawai) and B.t.k (Bacillus thuriengis subspecies kurstaki) in a co-culture system. The reason to co-cultivate B.t.a and B.t.k is to develop a combined formulation for endo-toxins produced by these two sub-species of B.t (for better pesticide activity). Similar morphology between B.t.a and B.t.k gave us hard time, determining the individual species dynamics in a co-culture system. The results were interesting and encouraging. However, after spending 18 months (I hardly closed my eyes during this time) on the project, working through the night, we were not able to publish the results due to logistics. This work was done at the International center for bioprocess technology, Anna University. Some industrial trainees (we lost Mr. Manivannan to brain fever during a field study and the news was shocking) from TUTICORIN Alkali Chemicals also played an active role in this work during 1994-1995. However, G. Kalaiselvi and R. Sachidanandam successfully completed the maintenance requirements in Bacillus sphaericus 1593M under dual substrate limitations estimated at zero growth rate in a total cell retention culture [15].

## Biotransformation of ibuprofen using Candida rugosa lipase:

During the summer of 1996, I started working on several aspects of lipase science and engineering. Industrial application of lipase is well known. It should be noted that lipase is a non Michaelis-Menten enzyme and it does not follow the Michaelis-Menten kinetics. Hence, discovery, optimization and large scale production of lipase is highly relevant to the society.

Large scale production of lipase is of social importance. Hence, we (P. Kangueane, P. Gautam, B.S. Lakshmi and B. Abraham) optimized lipase production by Candida rugosa using vegetable oils as substrates. Thus, the effect of vegetable oils in the secretion of lipase from Candida rugosa (DSM 2031) was optimized [16]. One of key challenges was lipase assay. Therefore, we (P. Kangueane, P. Gautam, B.S. Lakshmi and M. Krishnan) developed a simple, fast, sensitive assay method for Candida rugosa lipase using a bi-phasic reaction system. This was explained by the solvent hydrophobicity in the interfacial activation of Candida rugosa lipase [17]. The application of lipase for Biotransformation of a NSAID ibuprofen was exceedingly imperative. Consequently, we (P. Kangueane, P. Gautam, B.S. Lakshmi, Y. Gao and Y.Z. Chen) showed the stereo-specificity of S(+) ibuprofen to Candida rugosa. Then, the molecular basis for the stereo-specificity of Candida rugosa lipase (CRL) towards ibuprofen is shown [18]. It is well known that enzymes are both substrate specific and stereo-specific in nature. However, there is no material evidence to validate this realization. Hence, we (J James, B.S. Lakshmi, P. Gautam, P. Kangueane) showed the flap movement in different pH conditions using molecular dynamics simulation. Consequently, an insight from molecular dynamics simulations into pH-dependent enantioselective hydrolysis of ibuprofen esters by Candida rugosa lipase is documented [19].

## Bioinformatics at National University of Singapore:

The Bioinformatics Centre (BIC) at the National University of Singapore was founded by Tan Tinwee with the help of S Subbiah. Tan Tinwee is a visionary and his passion for internet networking is tremendous. S. Subbiah is the author of multiple sequence alignment and side chain packing for protein modeling. His contribution to Bioinformatics is highly significant. The contributions made by Prasanna Kolatkar in protein crystallography in the region are considerable. The application of Standard Markup Language (SML) in Bioinformatics by Wong Limsoon is highly commented.

## From HLA-peptide binding prediction to peptide vaccine design:

Short antigen peptides capable of binding host HLA molecules can be used to design peptide vaccines by exploiting T-cell immunity. Two problems with the design of such a cocktail vaccine are antigen peptide diversity from viral/bacterial proteome and host HLA allele polymorphism. The key issue is specific HLA-peptide binding. This is rate limiting in T-cell mediated immune response. Therefore, HLA-peptide binding prediction is highly relevant. Hence, we (P. Kangueane, M.K. Sakharkar, E.C. Ren and P.R. Kolatkar) developed a method to predict peptides binding to HLA molecules using side chain packing molecular modeling techniques developed by S. Subbiah. This is done using the knowledge-based grouping of modeled HLA peptide complexes [20]. The ranking of modeled complexes on the basis of van der Waals clash is promising. It should be noted that S. Subbiah made several generous contributions towards this study. Betty Cheng gave her ORIGIN SGI machine to perform the modeling calculation and data storage. Her generosity is highly appreciated. Large scale application of HLA-peptide model binding evaluation was eminent. Therefore, we (E.C. Ren, P. Kangueane and P.R. Kolatkar) studied the binding of mHag (minor histo-compatibility antigen involved in graft versus host disease) peptides to HLA A alleles. Thus, molecular modeling of the minor histocompatibility antigen HA-1 peptides binding to HLA-A alleles was insightful [21].

The urge to improve HLA-peptide binding prediction is evolving. This is possible by understanding the molecular principles of HA-peptide binding. Hence, we (P. Kangueane, M.K. Sakharkar, E.C. Ren and P.R. Kolatkar) studied the structural principles of HLA-peptide binding using a dataset of HLA-peptide crystal structures. Thus, the path towards the MHC-peptide combinatorics scanning is well established [22]. This is exemplified by the type of inter-atomic interactions at the interfaces of HLA-peptide structures using a dataset (Adrian et al. 2002). The types of inter-atomic interactions at the MHC-peptide interface by identifying commonality from accumulated data provided valuable insights [23]. This is biological knowledge discovery in computational immunology. So, data, dataset and database are the key to biological knowledge discovery. Consequently, T.W. Tan, R. Shoba and Govindarajan helped to develop the MPID database [24]. The MPID: MHC-Peptide Interaction Database for sequence-structure-function information on peptides binding to MHC molecules was created at an appropriate juncture. Further, Bing Zhao helped to develop methods to compress functional diversity among HLA alleles by listing common features among HLA alleles. Thus, compression of functional space in HLA-A sequence diversity was insightful [25]. Eventually, these discoveries in Immuno Informatics analysis helped to develop a novel method to predict HLA-peptide binding by Zhao et al. 2003 [26]. Moreover, the class II HLA-peptide binding prediction using structural principles was completed by Mohanpriya et al. 2009 [27]. This later helped us (P. Kangueane, P. Shapshap, S. Subbiah) to subtype HLA super-types (functional overlap among alleles) in 2005. Eventually, a framework to sub-type HLA supertypes is visualized [28]. This is further supported by a HLA-DRB supertype chart with potential overlapping peptide binding function [29]. The structural basis for HLA-A2 supertypes [30] provided adequate explanation for the phenomenon. The grouping of class I HLA alleles using electrostatic distribution maps of the peptide binding grooves [31] added clarity to the HLA-petide binding. Thus, HLA-peptide binding prediction using structural and modeling principles is reliable and feasible [32]. Furthermore, the structure modeling based computer aided T-cell epitope design is highly possible [33]. These advancements created the T-Epitope Designer: A HLA-peptide binding prediction server [34]. Accordingly, designing short peptides as viral vaccines is promising [35]. We then demonstrated the utility of this technology in the design of a gp120 peptide vaccine cocktail vaccine for NeuroAIDS in a book chapter edited by Karl Goodkin [36]. The designing of HIV gp120 peptide vaccines: rhetoric or reality for neuroAIDS is thus illustrated. Development of HIV-1/AIDS vaccine is non-trivial. An analysis of HIV-1 envelope accessible surface and polarity: clade, blood, and brain are relevant [37] in the context of NeuroAIDS [38] - [41]. These developments lead to the creation of the book titled Global Virology III: Virology in the 21st Century [79]. In this context, the availability of emerging technologies for anti-viral drug discovery is pertinent [81].

## Protein-Protein interactions:

Protein-Protein interactions [75], [76], [78] play an important role in catalysis, regulation and immune response. Therefore, it is of important to understand the molecular principles of protein-protein interaction using protein structure complexes. During the summer of 1995, I had an opportunity to work on the principles of protein-protein interaction at the labs of P. Balaram and C. Ramakrishnan using a dataset of protein structural complexes. I started working with K. Gunasekaran to understand protein subunit principles at the Molecular Biophysics Unit, Indian Institute of Science using a software program named merint.f. C. Ramakrishanan was the author of a FORTRAN program “merint.f “that calculates inter atomic distance between two polypeptide chains. The initial part of this work was done at the Molecular Biophysics Unit, IISC, India (1995) and later part was done at NTU, Singapore (2002-2006) and VIT University (2007-2009) and AIMST University, Malaysia (2009-2011). Protein-protein interfaces are hydrophobic. Protein subunit interfaces are either heterodimers or homodimers [42]. Protein-protein interfaces are vdW dominant with selective H-bonds and (or) electrostatics towards broad functional specificity [43]. Hot spot residues at protein-protein interface are relevant [44]. Interaction modes at protein hetero-dimer interfaces are insightful [45].Critical heterodimer protein interface parameters by multi-dimensional scaling in euclidian space were known [46]. Insights from the structural analysis of protein heterodimer interfaces are useful [47]. However, small protein-protein interfaces rich in electrostatic are often linked to regulatory function [48].

Homodimer protein interfaces are intriguing. The structural features for homodimer folding mechanism are insightful [49]. Moreover, structural features differentiate the mechanisms between 2S (2 state) and 3S (3 state) folding homodimers [50]. A decision tree model for the prediction of homodimer folding mechanism is useful [51]. A CART assignment of folding mechanisms to homodimers with known structures is worthy [52]. A dataset of ligand effect on homodimer interface was meaningful [53]. Structural inferences for cholera toxin mutations in Vibrio cholera are valuable [54].

## Genome analysis:

Genome analysis was imperative in 2000 with the completion of the human genome project. The availability of the genome data provided impetus to analyse gene fusion and study gene architecture across genomes.

Gene fusion is a phenomenon that has generated much curiosity since its description. Human fusion proteins are found to mimic operons and protein-protein interfaces in prokaryotes. They are also found to exhibit multiple functions and alternative splicing. We started digging deep for human fusion genes of prokaryotic origin [55][55]. This provided insights to metabolic network evolution by fusion proteins [56]. This is further explained by insights on gene fusion from molecular dynamics simulation of fused and un-fused IGPS (Imidazole Glycerol Phosphate Synthetase) [57][57]. A database on alternatively spliced human genes by exon skipping named ASHESdb was striking [58][58]. 

The ExInt (Exon-Intron) database was generated using GenBank feature. The GenBank feature CDS was used to create the IE-Kb: intron exon knowledge base [59]. Intron position conservation across eukaryotic lineages in tubulin genes was illustrated [60]. The distribution of exons and introns in the human genome was realized [61]. An analysis on gene architecture in human and mouse genomes was insightful [62].

The SEGE: A database on 'intron less/single exonic' genes from eukaryotes [63] was further developed using GenBank. The Genome SEGE: a database for 'intronless' genes in eukaryotic genomes was created from Genome databases [64]. A report on single exon genes (SEG) in eukaryotes [65] with computational prediction of SEG (single exon gene) function in humans [66] was representative in nature.

The human genome illustrated from pieces to patterns was graphical [67]. The u-Genome: a database on genome design in unicellular genomes is intriguing [68]. The presence of huge proteins in the human proteome and their participation in hereditary diseases is insightful [69].

## Bioinformation at Biomedical Informatics:

Creating literature is critical. Biological knowledge discovery beyond Bioinformatics [73], [80] is highly imperative in modern medicine. Bioinformation Discovery [74], [77] from Biological data using Bioinformatics Tools and analysis has become a routine procedure in Biological knowledge discovery. Thus, the formation and development of Bioinformation [70], an open access (free to read) journal in Biology is appropriate to the scientific community. Access to available literature for advancement through the application of science for the society is specifically complex. The quote from BOAI "the promise was that removing access barriers would allow the world to "accelerate research, enrich education, share the learning of the rich with the poor and the poor with the rich... and lay the foundation for uniting humanity in a common intellectual conversation and quest for knowledge" explains everything. This is often non-trivial [71] - [72] [71-72]. There are several challenges linked to this noble cause. 

“Linking, collaborations, sharing of data, reading, writing and editing among the literate community are the way of life in modern civilization”

In an active lifetime of a scientist

This work is dedicated to mother Muthalammale
